# Trends in stroke occurrence in rheumatoid arthritis: a retrospective cohort study from Western Norway, 1972 through 2020

**DOI:** 10.3389/fmed.2025.1547518

**Published:** 2025-05-21

**Authors:** Christian Lillebø Alsing, Jannicke Igland, Tone Wikene Nystad, Clara Gram Gjesdal, Halvor Næss, Grethe S. Tell, Bjørg-Tilde Fevang

**Affiliations:** ^1^Department of Internal Medicine, Haraldsplass Deaconess Hospital, Bergen, Norway; ^2^Department of Clinical Science, University of Bergen, Bergen, Norway; ^3^Department of Heart Disease, Haukeland University Hospital, Bergen, Norway; ^4^Department of Global Public Health and Primary Care, University of Bergen, Bergen, Norway; ^5^Department of Health and Social Science, Centre for Evidence-Based Practice, Western Norway University of Applied Science, Bergen, Norway; ^6^Department of Rheumatology, Haukeland University Hospital, Bergen, Norway; ^7^Department of Neurology, Haukeland University Hospital, Bergen, Norway; ^8^Centre for Age-Related Medicine, Stavanger University Hospital, Stavanger, Norway

**Keywords:** rheumatoid arthritis, cardiovascular, epidemiology, observational, comorbidity/multimorbidity, stroke

## Abstract

**Objectives:**

To investigate stroke occurrence in patients with rheumatoid arthritis (RA) diagnosed from 1972 through 2013 compared with the total population.

**Methods:**

We included 1821 patients diagnosed with RA from 1972 through 2013 at Haukeland University Hospital in Norway. The patients were divided into three inception cohorts by time of RA diagnosis (1972–1998, 1999–2006, and 2007–2013), based on major changes in RA treatment. The total population of the same county was used as a comparison cohort. Both cohorts were followed from 1972 through 2020. Annual change in stroke event rates were calculated by Poisson regression with adjustment for age and sex. Hazard ratios were estimated by Cox regression with adjustment for age, sex, smoking, BMI, diabetes and serological status. Standardized event ratios (SER) were estimated by Poisson regression as a measure of excess stroke events in patients with RA compared with the total population.

**Results:**

In total 156, 70, and 31 stroke events occurred in the first, middle, and last inception cohorts during, respectively, 17,110, 9,561, and 4,098 person-years of observation. From 1999 to 2020 stroke event rates declined by 4.8% (95% CI 2.7–6.9) per year in the RA cohort and by 3.4% (95% CI 3.1–3.7) per year in the comparison cohort. There was a trend towards lower stroke risk across inception cohorts, with a statistically significant reduction only observed among women in the last cohort compared to the first cohort (hazard ratio 0.30, 95% CI 0.12–0.76). Despite this reduction, the last inception cohort had the highest excess of stroke events (SER 1.58, 95% CI 1.03–2.43), compared with the total population. However, this excess stroke occurrence was only observed in men with RA and not in women with RA.

**Conclusion:**

Despite an overall decline in stroke occurrence over time, men with RA diagnosed after 2007 had a residual excess stroke occurrence compared to the total population. No excess stroke occurrence was observed in women with RA from the same time period. Our findings highlight the continued need of targeted stroke prevention in patients with RA, particularly for men.

## 1 Introduction

Rheumatoid arthritis (RA) is the most common chronic inflammatory joint disease, affecting 0.5–1% of the population (1). Previous studies have shown an increased risk of cardiovascular disease (CVD) in RA compared with the general population and patients with RA have a 60% increased risk of stroke (2), possibly higher in young and anti-citrullinated protein antibodies (ACPA) positive patients (3–5).

Excess CVD in RA is caused by a combination of traditional CVD risk factors and systemic inflammation. Among the known risk factors, smoking is highly prevalent in patients with RA, and hypertension is often undertreated (6, 7). Inflammation is known to accelerate atherosclerosis, while specific anti-inflammatory treatment prevents cardiovascular events (8). In patients with RA, high disease activity correlates with high CVD risk (9, 10), and remission, on the other hand, is associated with lower CVD risk (11, 12). Furthermore, atrial fibrillation, a common cause of stroke, has been linked to inflammation and is more frequent in patients with RA compared with the general population (13).

RA management has improved significantly during the 21st century. The two most important milestones are arguably the introduction of biologic disease-modifying anti-rheumatic drugs (DMARDs) in 1998 and the treat-to-target strategy from 2004 and onwards (14). With modern treatment, a higher proportion of patients achieve remission, and consequently fewer experience joint destruction leading to less joint replacement surgery (15). Several observational studies report lower CVD and stroke risk in patients with RA treated with methotrexate and biological DMARDs (16, 17).

Recent studies on CVD in RA diagnosed during the 21st century show conflicting results. Both Løgstrup and Baviera have found a persistent excess stroke occurrence in patients with RA diagnosed during 1997–2017 and 2005–2017 compared with the general population (18, 19). Other studies, however, have found a decline or no excess stroke occurrence in patients with RA diagnosed during equivalent time periods (20, 21). Several studies have found a persistence of excess myocardial infarction in patients with RA compared with the general population (22–24). However, other studies, including a study published by our research group, found no excess occurrence of myocardial infarction in patients with RA diagnosed in the 21st century (25, 26).

Few studies have investigated stroke occurrence in relation to the time of RA diagnosis, comparing before and after the major improvements in RA treatment. We therefore aim to investigate stroke occurrence in patients with RA diagnosed during different treatment eras compared with the general population.

## 2 Materials and methods

### 2.1 Study design and settings

We performed a retrospective cohort study of 1821 patients with RA diagnosed at the Department of Rheumatology, Haukeland University Hospital during 1972–2013. There are currently only 2 rheumatologists in private practice in the county, hence almost all patients with RA are treated at the hospital. The total population of the same county (Hordaland) was used as a comparison cohort. Individual data were available for the RA cohort, while data for the comparison cohort were available as aggregated counts by 5-year age group, sex, and calendar year. Both cohorts were followed from 1972 through 2020.

The study complies with the Declaration of Helsinki and the STROBE (Strengthening the Reporting of Observational Studies in Epidemiology) statement and was approved by the Regional Committee for Medical and Health Research Ethics, Health Region West (2014/1932) and the institutional review board (27).

### 2.2 Patients with RA

Full details of the selection and inclusion of patients with RA, including a flowchart and a comparison of included and excluded participants, have been published previously (24). In summary, we searched the hospital patient administrative system for patients with an outpatient contact or hospital admission with an RA diagnosis during 1972–2013 at the Department of Rheumatology, Haukeland University Hospital. Then, consent for inclusion was acquired either by letter of consent or through the Norwegian Arthritis Registry. Exemption of consent was allowed for deceased patients (*n* = 979). The medical records of the included individuals were reviewed by two physicians. RA was defined as present if diagnosed by the treating rheumatologist, and this was mandatory for inclusion. Participants were excluded if the final diagnosis during follow-up was not RA. Data on characteristics of patients with RA (Table 1) were derived from the medical records.

**Table 1:** Characteristics of 1821 patients with rheumatoid arthritis (RA), Hordaland County, Norway 1972–2013.

Characteristics	Time of RA diagnosis		
	1972–1998 (*N* = 771)	1999–2007 (*N* = 642)	2008–2013 (*N* = 408)
Women, *n* (%)	559 (72.5%)	445 (69.3%)	261 (64.0%)
Age at RA diagnosis, years	54 (15.4)	56.2 (16.1)	55.8 (15.5)
BMI^a^, kg/m^2^; mean (SD)	24.9 (4.3)	25.5 (4.5)	25.7 (4.4)
Missing, *n* (%)	77 (10.0%)	43 (6.7%)	1 (0.3%)
Smoking status at RA diagnosis			
Missing, *n* (%)	45 (5.8%)	3 (0.5%)	1 (0.2%)
Non-smoker, *n* (%)	372 (51.2%)	264 (41.3%)	161 (39.6%)
Former smoker, *n* (%)	121 (16.7%)	198 (31.0%)	141 (34.6%)
Smoker, *n* (%)	233 (32.1%)	177 (27.7%)	105 (25.8%)
Comorbidities^b^			
Previous AMI, *n* (%)	36 (4.7%)	35 (5.5%)	19 (4.7%)
Previous stroke, *n* (%)	14 (1.8%)	22 (3.4%)	11 (2.7%)
Diabetes, *n* (%)	20 (2.6%)	38 (5.9%)	30 (7.4%)
Angina, *n* (%)	50 (6.5%)	40 (6.2%)	17 (4.2%)
Antihypertensive drug use, *n* (%)	88 (11.4%)	141 (22.0%)	106 (26.0%)
Statin use, *n* (%)	8 (1.0%)	89 (13.9%)	42 (10.3%)
ACR/EULAR criteria fulfilled^c^, *n* (%)	654 (84.8%)	563 (87.7%)	370 (90.7%)
RF/ACPA positive^a^, *n* (%)	480 (62.4%)	418 (65.2%)	301 (73.8%)
ESR^d^, mm/h; mean (SD)	51.4 (31.5)	46.4 (26.1)	39.4 (25.1)
CRP^d^, mg/l; mean (SD)	44.4 (44.4)	42.8 (47.3)	30.0 (37.6)
Missing, *n* (%)	300 (39%)	1 (0%)	0 (0%)
Involvement of large joints, *n* (%)	546 (70.8%)	441 (68.7%)	232 (56.9%)
Radiographic erosions^a^, *n* (%)	577 (76.3%)	288 (46.1%)	137 (33.7%)

ACPA, anti-citrullinated protein antibodies; ACR, American College of Rheumatology; AMI, acute myocardial infarction; BMI, body mass index; CRP, c-reactive protein; DMARD, disease-modifying anti-rheumatic drug; ESR, erythrocyte sedimentation rate; EULAR, European Alliance of Associations for Rheumatology; NSAID, non-steroidal anti-inflammatory drug; PVD, peripheral vascular disease; RA, rheumatoid arthritis; RF, rheumatoid factor; SD: standard deviation.

a During follow-up. BMI was calculated using the available height and weight measurements from medical records nearest in time to RA diagnosis.

b Before and 1 year after RA diagnosis.

c The 2010 ACR/EULAR criteria were used for all patients.

d The highest value within 1 year before and 2 years after the diagnosis of RA.

Patients with RA were divided into three inception cohorts by year of RA diagnosis: 1972–1998, 1999–2007, and 2008–2013. The chosen time periods reflect improvements in RA management: in 1998 the introduction of biological DMARDs; in 2007 the universal adoption of the treat-to-target strategy at our department.

### 2.3 Comparison cohort

All inhabitants in Hordaland County (378,261 in 1972 to 524,495 in 2019), including patients with RA, were used as a comparison cohort. Aggregated counts of the population per calendar year, 5-year age group, and sex were obtained from the Western Norway Cardiovascular Registry (WENOCARD) (1972–2006) and the Cardiovascular Diseases in Norway project (CVDNOR) (2007–2014). During 2015–2020 population counts were obtained from Statistics Norway. WENOCARD and CVDNOR have been described in detail previously (25, 28, 29). We did not have additional data on cardiovascular risk factors for the comparison cohort.

### 2.4 Outcomes

A stroke event was defined as a hospitalization with stroke (ICD-8; 430, 433, 431, 434, 436, ICD-9; 430, 431, 434, 436 and ICD-10; I60, I61, I63, I64) as a primary or secondary diagnosis. Recurrent events were counted if more than 28 days passed between discharge and the next admission. An incident event was defined as the first hospitalization with stroke as a primary or secondary diagnosis after RA diagnosis.

Data on stroke events during 1972–2006 were obtained from WENOCARD for both patients with RA and the comparison cohort. Outcomes were obtained from CVDNOR (2007–2014) and the Norwegian Cardiovascular Disease Registry (NCDR) (2015–2020) for the comparison cohort and by review of hospital patient administrative systems (2007–2020) for the RA cohort. The data from CVDNOR and NCDR both originate from discharge codes in the hospitals’ patient administrative systems.

### 2.5 Statistical analysis

Characteristics of patients with RA were compared across three inception cohorts (Table 1) and furthermore stratified by sex (Supplementary Tables 1 and 2). The time of RA diagnosis was defined as the first of January in the year RA was diagnosed by the treating rheumatologist. The follow-up time used to count stroke events was defined as the time between the time of RA diagnosis and death or the end of the study (31st of December 2020). However, for calculating incidence rates and time-to-event analyses, the study end was extended to 31^st^ December, 2022, due to supplementary RA cohort data. Continuous variables are presented as means with standard deviations and categorical variables as numbers and proportions. Missing data are reported when present in more than 2% of the inception cohort. Welch’s t-test was used to test for statistically significant differences between groups for continuous variables and chi-square test for categorical variables.

Crude rates of stroke incidence and events were estimated per year for the RA cohort and comparison cohort to delineate changes from 1972 to 2022. Cubic splines with 3 knots were fitted to show trends due to fluctuations in stroke rates in the RA cohort. Annual age-adjusted stroke event rates were calculated using direct standardization, with the 2013 European Standard Population as the reference population. This analysis was restricted to the comparison cohort due to lack of statistical power in the RA cohort.

Annual changes in event and incidence rates were estimated by Poisson regression models with calendar year as a continuous predictor and person-time as an offset. Separate estimates were calculated for 1972–2020 and 1999–2020 to address the biphasic trend in observed stroke rates in both cohorts due to secular changes in the definition and diagnosis of stroke. The exponentiated coefficient for calendar year represents the incidence rate ratio (IRR) corresponding to the average annual relative change in incidence rates. The annual change can be calculated by subtracting the IRR from one. The analyses were performed unadjusted and adjusted for age, sex, BMI, diabetes, smoking and serological status.

Time-to-event analyses were performed for the RA cohort to investigate stroke incidence. Patients with stroke before RA diagnosis were excluded from these analyses. We created cumulative incidence functions adjusted for competing risk of death prior to stroke. Hazard ratios were estimated using Cox regression with inception cohorts as a categorical predictor, adjusting for age, sex, smoking, BMI, diabetes and serological status. The proportional hazards assumption was verified by statistical testing of Schoenfeld residuals.

We estimated standardized event ratios (SER) as a measure of excess stroke events in patients with RA compared with the general population for each inception cohort and across subgroups. Reference rates were calculated per 5-year age group, sex, and calendar year using counts of stroke events and mid-year population at risk in the comparison cohort. Counts of stroke events in the RA cohort were aggregated according to the same strata as the comparison cohort. We then calculated the expected counts of stroke events for each stratum in the RA cohort using the population at risk and the reference rates. The expected counts were included as an offset in a Poisson model with counts of stroke events as the dependent variable and standardized event ratios as the output. All Poisson regression analyses are reported using robust 95% confidence intervals to adjust for overdispersion. There were no missing data in the regression analyses. Sensitivity analyses were performed setting the baseline to 1^st^ of January 1 year after RA was diagnosed.

Analyses were conducted using Stata Statistical Software (release 17 or later; StataCorp LP, College Station, TX) and R (version 3.6.3 or later). A *p*-value below 0.05 was considered significant and the *p*-values are not adjusted for multiple testing.

## 3 Results

Three thousand and seventy-six patients were identified using the electronic patient administrative system. Four hundred and twenty-nine patients did not consent to inclusion. Among consenting individuals, eight hundred and twenty-eight patients were excluded mainly due to incorrect RA diagnosis (*n* = 279), incomplete medical records (*n* = 282), receiving follow-up by a private practicing rheumatologist (*n* = 89) or being diagnosed with RA prior to 1972 or after 2013 (*n* = 178). Thus, a total of 1821 patients with RA were included in this study across three inception cohorts according to the year of RA diagnosis: 771 patients during 1972–1998; 642 patients during 1999–2007 and 408 patients during 2008–2013. The comparison cohort included on average 325,537, 394,509 and 437,656 inhabitants during 1972–1998, 1999–2007 and 2008–2020, respectively.

### 3.1 Patient characteristics

Patients with RA in the two latest inception cohorts were on average older at baseline, had higher BMI, more frequently diabetes, and were more likely to use antihypertensives or statins (Table 1) compared with patients diagnosed before 1999. Manifestations of radiographic erosions during follow-up were more prevalent in the first inception cohort (76.3%) compared with the later inception cohorts (respectively 46.1 and 33.7%). Patients with RA diagnosed in the last inception cohort had the lowest levels of ESR or CRP at baseline. Smoking prevalence declined from 32 to 25.8% from the first to last inception cohort.

Men and women had similar characteristics across inception cohorts (Supplementary Tables 1 and 2), except for persistently lower smoking rates (respectively 33 and 26%) and lower levels of diabetes (respectively 7.7 and 3.6%) in women. Comparing the middle inception cohort to the latest cohort revealed several changes specific to women: mean CRP levels decreased significantly (mean difference 16, 95% CI 9.81–22.15, *p* < 0.001); the prevalence of radiographic arthritis decreased from 49.4% in the middle cohort to 31.5% in the latest cohort (*p* < 0.001); angina at baseline decreased from 6.1% in the middle cohort to 1.9% in the latest (*p* = 0.018). These particular characteristics did not significantly differ between the middle and latest cohorts among men. Lastly, DMARD use within the first year of RA was similar among men and women, although a higher proportion of men received biologic DMARDs in the latest cohort (10.3% of men vs. 5.4% of women).

### 3.2 Stroke incidence and time-to-event analyses

**Figure 1:**
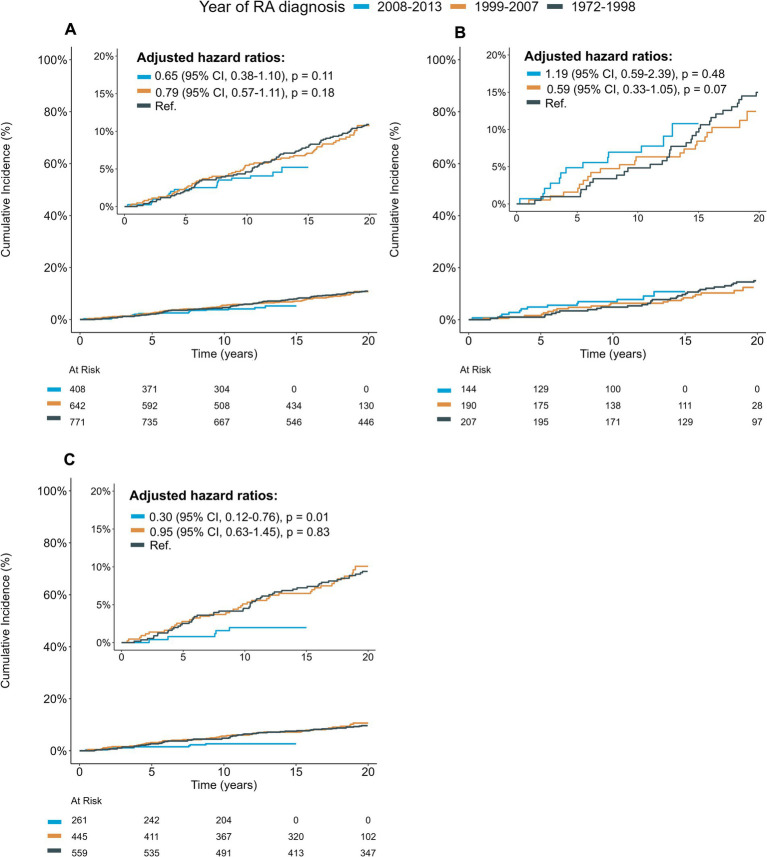
Cumulative incidence functions showing time to first stroke hospitalization for the total RA cohort **(A)**, for men **(B)**, and women **(C)** stratified by time of RA diagnosis. The cumulative incidence was adjusted for competing risk of death. Patients with stroke prior to RA diagnosis were excluded from the analyses. Reported hazard ratios were estimated by cox regression, with adjustment for age, sex, smoking, BMI, diabetes and serological status. CI; Confidence interval; RA, rheumatoid arthritis.

Annual stroke incidence declined on average 3.2% (95% CI 0.8–5.6) in the RA cohort, after adjusting for sex, age, BMI, diabetes, smoking and serological status (Supplementary Table 3). The 20-year cumulative incidence of stroke was similar in the first (10.9%) and middle (10.8%) inception cohorts (Figure 1). However, there was a trend towards lower stroke incidence in the latest inception cohort, diverging from earlier cohorts from 5 years after RA diagnosis and onwards. There was no statistically significant difference in stroke risk compared to the first cohort (HR 0.65, 95% CI 0.38–1.11, *p* = 0.11) after adjusting for age, sex, smoking, BMI, diabetes and serological status. In contrast, stratified analysis by sex revealed a significantly lower stroke risk among women in the latest cohort (HR 0.30, 95% CI 0.12–0.76, *p* = 0.01), but no significant difference for men (HR 1.19, 95% CI 0.59–2.39, *p* = 0.48).

### 3.3 Stroke events

In total, 156 stroke events occurred in 109 patients diagnosed with RA during 1972–1998 over 17,110 person-years of follow-up, 70 stroke events occurred in 55 patients with RA diagnosed during 1999–2007 over 9,561 person-years of follow-up and 31 stroke events occurred in 20 patients with RA diagnosed during 2008–2013 over 4,098 person-years of follow-up. In the comparison cohort 21,210, 12,152 and 14,451 stroke events occurred during, respectively, 1972–1998, 1999–2007 and 2008–2020.

The overall temporal trend for crude event rates of stroke appeared biphasic for both the comparison and RA cohort, declining from 2000 and onwards, likely reflecting shifts in case definition and diagnosis over time (Supplementary Figure 1). From 1999 and onwards there was an annual decline of 4.8% (95% CI 2.7–6.9, *p* < 0.001) in age- and sex-adjusted stroke rates in the RA cohort, and 3.4% (95% CI 3.1–3.7, *p* < 0.001) in the comparison cohort (Supplementary Table 3).

**Figure 2:**
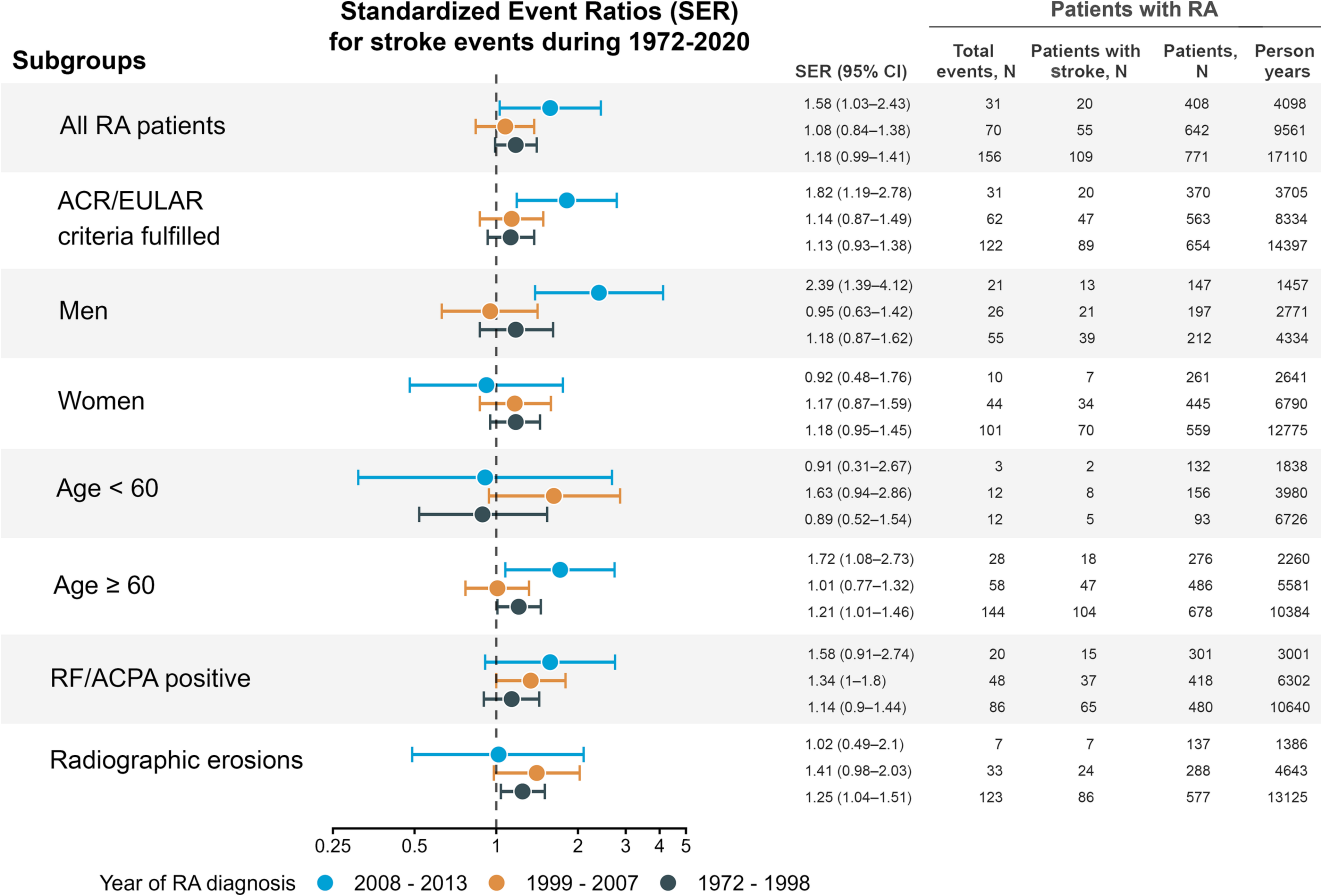
Standardized event ratios comparing stroke events in rheumatoid arthritis (RA) patients with the general population. Only inhabitants of Hordaland, Norway were included in the estimates. Point estimates are given with 95% robust confidence intervals. Separate estimates were calculated for the entire RA cohort and 6 RA subgroups defined by age, sex, positive rheumatoid factor or ACPA, fulfilment of the 2010 ACR/EULAR criteria, and radiographic erosions during follow-up. Counts of events, persons with stroke, and person-years of follow-up are shown only for the RA cohort, not the total population. Recurrent events were counted if more than 28 days between discharge to next admission. Person-years were estimated from the year of RA diagnosis to death or 31st December 2020. ACPA, anti-citrullinated protein antibodies; ACR, American College of Rheumatology; EULAR, European Alliance of Associations for Rheumatology; RA, rheumatoid arthritis; RF, rheumatoid factor; SER, standardized event ratio.

There was a general tendency of a moderate excess of stroke events across the RA subgroups (Figure 2). However, only patients with RA in the latest inception cohort had a statistically significant excess occurrence of stroke events (SER 1.58, 95% CI 1.03–2.43) compared with the total population and adjusted for year of event, sex, and age group. The excess exclusively observed in the male RA subgroup (SER 2.39, 95% CI 1.39–4.12), and no statistically significant excess was observed for female RA patients.

In the first inception cohort, we found an excess occurrence of stroke events of 18% (SER 1.18, 95% CI 0.99–1.41, *p* = 0.066), compared with the total population. Some subgroups of the first inception cohort had a significant excess of stroke events: patients older than 60 years (SER 1.21, 95% CI 1.01–1.46) and those with radiographic erosions (SER 1.25, 95% CI 1.04–1.51). In the middle inception cohort, only the RF/ACPA positive subgroup had a significant excess occurrence of stroke events (SER 1.34, 95% CI 1.00–1.80, Figure 2). Sensitivity analyses (Supplementary Table 4), revealed similar results, although a slightly lower estimate of excess stroke occurrence in the latest inception cohort.

## 4 Discussion

In this study, we compared stroke occurrence in patients with RA with the total population in the Hordaland municipality, Western Norway, stratified by the time period of RA diagnosis and adjusted for age, sex, and calendar year. We found a decline in stroke events over time in both the RA and comparison cohorts. However, a significant excess of stroke events was observed in male patients with RA, diagnosed between 2008 and 2013, but not in female patients. There was a trend towards excess stroke occurrence in patients diagnosed during 1972–1998, although not statistically significant and with similar estimates for men and women.

Surprisingly the most recent inception cohort had the highest estimated excess stroke occurrence compared with the general population. This excess stroke occurrence, however, was exclusively observed in male patients with RA, with no corresponding excess in female patients. This sex-specific discrepancy aligns with findings by Kerola et al., who found that RA conferred a significant stroke risk primarily in men, despite adjustment for cardiovascular risk factors (30).

Our study extends these observations by looking at temporal patterns. Unlike the study by Kerola et al., who only examined a contemporary RA cohort, our longitudinal analysis across multiple inception cohorts reveals that this discrepancy in stroke occurrence is a recent development, not mirrored in the general population. While the cause of this discrepancy remains unclear, this recent emergence suggests that the discrepancy is less likely driven by inherent biological sex differences. More likely, the disparities could be driven by non-biological factors, such as differences in health behaviors, including lifestyle factors, RA management, or possibly cardiovascular prevention.

Supporting these findings are the significant improvements in markers of RA disease activity across inception cohorts, specific to female patients with RA. Women in the latest inception cohort had lower average CRP, less radiographic arthritis, and less angina at baseline, and this trend did not extend to male patients with RA. These improvements occurred despite the observation of similar use of non-biological and even more frequent use of biological DMARDs in male patients with RA.

Notably, we found no significant differences between men and women regarding the use of statins or antihypertensives across inception cohorts. Disparities in the use of cardiovascular prevention are therefore an unlikely explanation for the observed sex-specific discrepancy.

Several factors could explain why the estimated excess was most prominent in the last inception cohort. Misclassification of stroke events likely occurred more frequently in earlier inception cohorts due to less accurate diagnostics, potentially obscuring excess stroke occurrence in these periods. Competing risk from higher mortality in RA could also influence our estimates, causing potentially lower estimates of stroke events in the earlier inception cohorts with longer follow-up periods.

Our findings of excess stroke occurrence in a contemporary RA cohort is in line with several previous studies. Løgstrup et al. recently found a 22% increased risk of stroke in RA diagnosed during 1996–2017 who were prescribed DMARDs during a 10-year follow-up period. Baviera et al. found a 39% increased risk of stroke in a RA cohort diagnosed during 2005–2017 identified from co-payment exemption codes (18, 19). Our study corroborates these findings by investigating patients with RA with a diagnosis established by a rheumatologist, not registry codes, which adds clinical value. We also compared the same RA cohort before and after improvements in RA treatment and investigated subgroups defined by RA characteristics, such as RF/ACPA-positive patients.

In contrast to our findings, Holmqvist et al. did not find excess stroke risk in an incident RA cohort diagnosed during 1997–2009 in Sweden (HR 1.11, 95% CI 0.95–1.30) (21). However, when stratified by RA disease duration, the risk was detectable after 10 or more years from RA diagnosis. Our study included stroke recurrence and patients with a stroke before RA diagnosis, while these were excluded from Holmqvist’s study. The follow-up period and period of RA diagnosis (1997–2009) overlapped in Holmqvist’s study, and therefore patients with RA with a diagnosis closer to the end of the study had a substantially shorter follow-up period than in our study.

Few previous studies have investigated stroke occurrence in RA inception cohorts over time compared with the general population. In accordance with our findings, Myasoedova et al. found a persistent excess stroke incidence in a study of 905 patients with RA diagnosed during 1980–2009 compared with 904 patients without RA stratified by the decade of RA diagnosis (26). Also, a retrospective cohort study from Spain found increasing stroke hospitalizations in patients with RA during 1999–2015 but did not compare with the general population (31). Our study corroborates these findings by investigating a larger RA cohort compared with the total population from the same geographical area.

In contrast to our findings, Yazdani et al. found a decline in ischemic stroke risk over time in a RA cohort diagnosed from 1999 up to 2004 compared with matched controls (20). Notably, they did not report sex-specific estimates. Several differences between our studies could explain the diverging results. Our study used the total population for comparison instead of matching, which is preferable to avoid possible selection bias. In addition, the definition of RA in our cohort was more stringent: We initially identified cases using ICD-codes, similar to Yazdani et al. but excluded 11% after a review of medical records finding a diagnosis of osteoarthritis or other inflammatory joint diseases than rheumatoid arthritis.

Our study has some limitations. We did not have data on traditional cardiovascular risk factors for the comparison cohort, and were subsequently unable to adjust the analyses on excess stroke occurrence for these confounders. Consequently the cause of the continued excess in stroke occurrence could not be established. The outcome included both hemorrhagic and ischemic stroke, which have different risk factors and mechanisms. We did not have data on race and ethnicity. Our cohort likely resembles that of the general Norwegian population, of which immigrants made up 17.3% of the total population in 2025, almost all of these from Nordic or European countries (32). Substantially fewer patients were included in the first RA inception cohort, which is likely due to an increase in referrals to secondary care along with improvements in RA management. Finally, we did not have data on incident events for the comparison cohort.

The key strengths of this study are the long follow-up period and the inclusion of patients diagnosed before and after improvements in RA treatment. Furthermore, patients with RA were compared with the total population instead of using a matched comparison cohort, which could lead to selection bias. The included patients also had an RA diagnosis of high certainty, and patients with other inflammatory joint diseases or osteoarthritis, who have a CVD risk different from that of patients with RA, were excluded from our cohort. Using registry or equivalent data sources also ensured the completeness of follow-up data and a high level of sensitivity in identifying stroke events.

## 5 Conclusion

Despite substantial declines in overall stroke rates over time, our findings suggest a residual excess stroke occurrence in patients with RA diagnosed after 2007 compared to the general population. Notably, this contemporary excess stroke burden was specific to male patients with RA. While the lack of excess stroke occurrence in women could reflect differences in benefits of improved RA management or cardiovascular care over time, our findings highlight the continued need of targeted stroke prevention for patients with RA, particularly in men.

## Data Availability

The datasets presented in this article are not readily available because the datasets analyzed for this study cannot be shared publicly since the approval by the regional ethics committee does not allow sharing of de-identified patient data. Aggregated data may be shared upon request, but further aggregation will be needed to ensure anonymity in the RA cohort. Requests to access the datasets should be directed to Christian Alsing, chr.alsing@gmail.com.
