# Supplementary material for: Trends in stroke occurrence in rheumatoid arthritis: a retrospective cohort study from Western Norway, 1972 through 2020
Source: Front Med (Lausanne). 2025 May 21;12:1547518. doi: 10.3389/fmed.2025.1547518 (PMC12133953; doi:10.3389/fmed.2025.1547518)
Supplement: Supplementary file 1 [file Data_Sheet_1.docx]

Supplementary Material

# Supplementary Figures and Tables

## Supplementary Figures


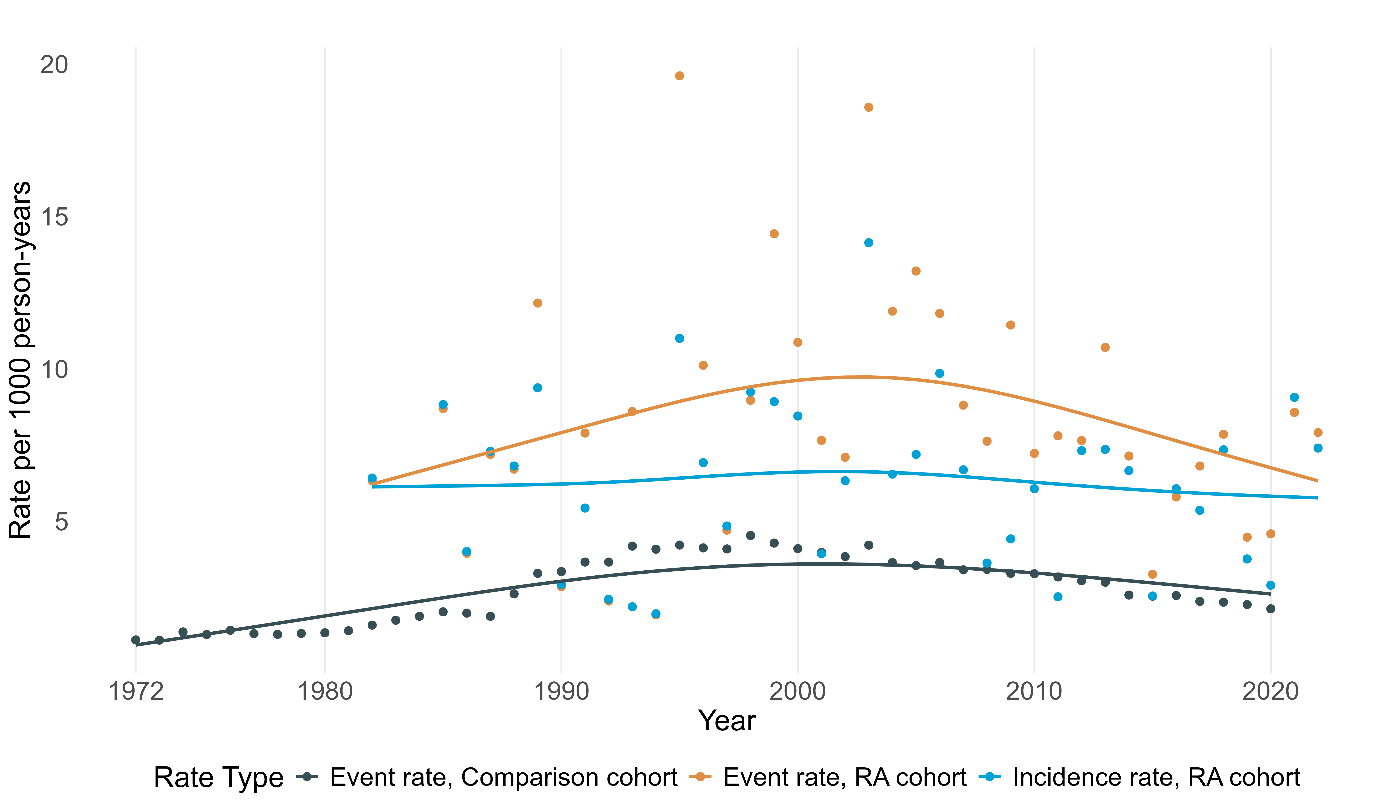


**Supplementary Figure 1.** Rates per year of incident strokes (blue) and stroke events in the RA and comparison cohort. The points represent observed data per year. Trends were modelled using cubic splines with 3 knots.
RA, rheumatoid arthritis.


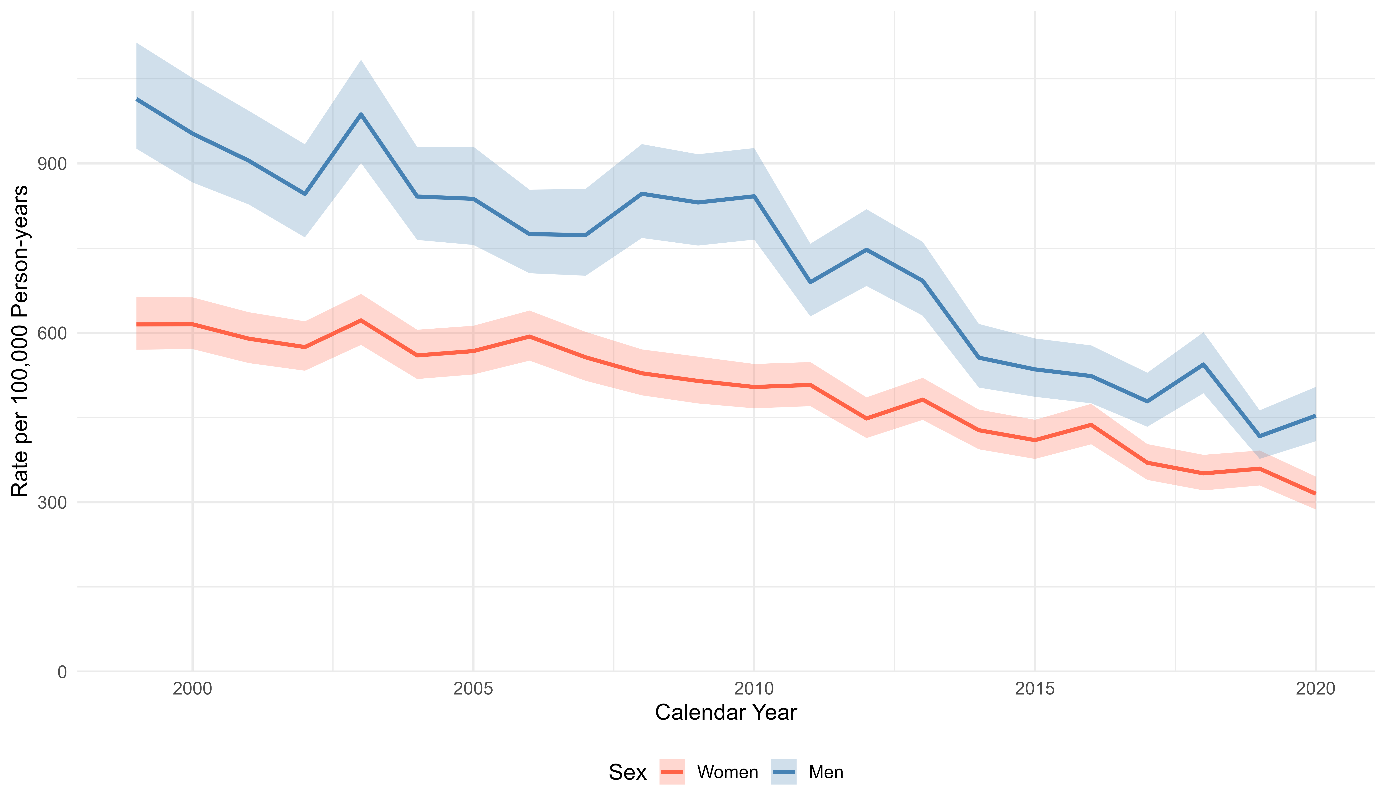


**Supplementary Figure 2:** Age-adjusted rates of stroke events in the comparison cohort per calendar year stratified by sex. Direct standardization was performed using the European Standard population.

## Supplementary Tables

| **Supplementary Table 1: Comparison of men with rheumatoid arthritis across inception cohorts.** | | | | |
| --- | --- | --- | --- | --- |
|  |  | Time of RA diagnosis | | |
| Characteristic | | 1972–1998  (N=212) | 1999–2007  (N=197) | 2008–2013 (N=147) |
| Age at diagnosis, Mean (SD) | | 55.4 (13.7) | 58.8 (14.6) | 56.9 (13.8) |
| BMI†, kg/m^2^, Mean (SD) | | 25.5 (3.8) | 25.9 (4.1) | 26.2 (3.9) |
| Smoking status, n (%) | |  |  |  |
| Smoker | | 86 (43.4%) | 55 (28.1%) | 44 (29.9%) |
| Non-smoker | | 62 (31.3%) | 59 (30.1%) | 36 (24.5%) |
| Former smoker | | 50 (25.3%) | 82 (41.8%) | 67 (45.6%) |
| Comorbidities* | |  |  |  |
| Previous AMI, n (%) | | 19 (9.0%) | 16 (8.1%) | 13 (8.8%) |
| Previous stroke, n (%) | | 4 (1.9%) | 8 (4.1%) | 4 (2.7%) |
| Diabetes, n (%) | | 10 (4.7%) | 17 (8.6%) | 16 (10.9%) |
| Angina, n (%) | | 20 (9.4%) | 13 (6.6%) | 12 (8.2%) |
| Antihypertensive treatment, n (%) | | 18 (8.5%) | 52 (26.4%) | 46 (31.3%) |
| Statin treatment, n (%) | | 5 (2.4%) | 27 (13.7%) | 17 (11.6%) |
| ACR/EULAR criteria, n (%) | | 171 (80.7%) | 182 (92.4%) | 134 (91.2%) |
| Affection of large joints, n (%) | | 156 (73.6%) | 154 (78.2%) | 99 (67.3%) |
| RF/ACPA positive†, n (%) | | 137 (64.9%) | 130 (66.0%) | 111 (75.5%) |
| ESR mm/h †, Mean (SD) | | 49.2 (31.3) | 47.3 (26.7) | 40.6 (27.0) |
| CRP mg/l †, Mean (SD) | | 48.8 (44.8) | 45.2 (40.7) | 37.6 (43.4) |
| Radiographic manifestations of arthritis†, n (%) | | 160 (76.6%) | 74 (38.5%) | 55 (37.4%) |
| DMARDs within first year of RA, n (%) | |  |  |  |
| None | | 32 (23.9%) | 26 (14.7%) | 14 (9.6%) |
| Other synthetic | | 77 (57.5%) | 24 (13.6%) | 8 (5.5%) |
| Methotrexate monotherapy | | 23 (17.2%) | 102 (57.6%) | 101 (69.2%) |
| Double/triple DMARD | | 2 (1.5%) | 9 (5.1%) | 8 (5.5%) |
| Biologic | | 0 (0.0%) | 16 (9.0%) | 15 (10.3%) |
| Missing | | 78 (37%) | 20 (10%) | 1 (0.7%) |

*Before and 1 year after RA diagnosis
†During follow-up. BMI was calculated using the available height and weight measurements from medical records nearest in time to RA diagnosis.
‡The highest value within 1 year before and 2 years after the diagnosis of RA
ACPA, anti-citrullinated protein antibodies; ACR, American College of Rheumatology; AMI, acute myocardial infarction; BMI, body mass index; CRP, c-reactive protein; DMARD, disease-modifying anti-rheumatic drugs; ESR, Erythrocyte sedimentation rate; EULAR, European Alliance of Associations for Rheumatology; NSAID, non-steroidal anti-inflammatory drugs; PVD, peripheral vascular disease; RA, rheumatoid arthritis; RF, rheumatoid factor.

| **Supplementary Table 2: Comparison of women with rheumatoid arthritis across inception cohorts.** | | | |
| --- | --- | --- | --- |
|  | Time of RA diagnosis | | |
| Characteristic | 1972–1998  (N=559) | 1999–2007  (N=445) | 2008–2013  (N=261) |
| Age at diagnosis, Mean (SD) | 53.5 (15.9) | 55.1 (16.6) | 55.2 (16.4) |
| BMI†, kg/m^2^, Mean (SD) | 24.7 (4.5) | 25.3 (4.7) | 25.4 (4.6) |
| Smoking status, n (%) |  |  |  |
| Smoker | 147 (27.8%) | 122 (27.5%) | 61 (23.5%) |
| Non-smoker | 310 (58.7%) | 205 (46.3%) | 125 (48.1%) |
| Former smoker | 71 (13.4%) | 116 (26.2%) | 74 (28.5%) |
| Comorbidities* |  |  |  |
| Previous AMI, n (%) | 17 (3.0%) | 19 (4.3%) | 6 (2.3%) |
| Previous stroke, n (%) | 10 (1.8%) | 14 (3.1%) | 7 (2.7%) |
| Diabetes, n (%) | 10 (1.8%) | 21 (4.7%) | 14 (5.4%) |
| Angina, n (%) | 30 (5.4%) | 27 (6.1%) | 5 (1.9%) |
| Antihypertensive treatment, n (%) | 70 (12.5%) | 89 (20.0%) | 60 (23.0%) |
| Statin treatment, n (%) | 3 (0.5%) | 62 (13.9%) | 25 (9.6%) |
| ACR/EULAR criteria, n (%) | 483 (86.4%) | 381 (85.6%) | 236 (90.4%) |
| Affection of large joints, n (%) | 390 (69.8%) | 287 (64.5%) | 133 (51.0%) |
| RF/ACPA positive†, n (%) | 343 (61.5%) | 288 (64.9%) | 190 (72.8%) |
| ESR mm/h ‡, Mean (SD) | 52.3 (31.6) | 46.0 (25.9) | 38.7 (24.0) |
| CRP mg/l ‡, Mean (SD) | 42.8 (44.2) | 41.7 (50.0) | 25.7 (33.3) |
| Radiographic manifestations of arthritis†, n (%) | 417 (76.2%) | 214 (49.4%) | 82 (31.5%) |
| DMARDs within first year of RA, n (%) |  |  |  |
| None | 66 (20.1%) | 81 (20.3%) | 28 (10.8%) |
| Other synthetic | 206 (62.8%) | 61 (15.3%) | 15 (5.8%) |
| Methotrexate monotherapy | 48 (14.6%) | 201 (50.4%) | 184 (70.8%) |
| Double/triple DMARD | 8 (2.4%) | 19 (4.8%) | 19 (7.3%) |
| Biologic | 0 (0.0%) | 37 (9.3%) | 14 (5.4%) |
| Missing | 231 (41%) | 46 (10%) | 1 (0.4%) |

*Before and 1 year after RA diagnosis
†During follow-up. BMI was calculated using the available height and weight measurements from medical records nearest in time to RA diagnosis.
‡The highest value within 1 year before and 2 years after the diagnosis of RA
ACPA, anti-citrullinated protein antibodies; ACR, American College of Rheumatology; AMI, acute myocardial infarction; BMI, body mass index; CRP, c-reactive protein; DMARD, disease-modifying anti-rheumatic drugs; ESR, Erythrocyte sedimentation rate; EULAR, European Alliance of Associations for Rheumatology; NSAID, non-steroidal anti-inflammatory drugs; PVD, peripheral vascular disease; RA, rheumatoid arthritis; RF, rheumatoid factor.

**Supplementary Table 3: Annual change in rates of stroke, 1972–2020 and 1999-2020.**

|  | RA cohort | |  | Comparison cohort |
| --- | --- | --- | --- | --- |
| **1972-2020** | Incidence rate | Event rate |  | Event rate |
| Unadjusted IRR^*^ | 1.000 (0.989-1.014) | 0.999 (0.987-1.011) |  | 1.011 (1.007-1.016) |
| Adjusted IRR† | 0.981 (0.967-0.994) | 0.976 (0.964-0.988) |  | 1.007 (1.005-1.009) |
| Fully adjusted IRR‡ | 0.979 (0.966-0.993) | - |  | - |
|  | RA cohort | |  | Comparison cohort |
| **1999-2020** | Incidence rate | Event rate |  | Event rate |
| Unadjusted IRR* | 0.983 (0.959-1.008) | 0.965 (0.941-0.990) |  | 0.968 (0.953-0.984) |
| Adjusted IRR† | 0.970 (0.947-0.994) | 0.952 (0.931-0.973) |  | 0.966 (0.963-0.969) |
| Fully adjusted IRR‡ | 0.968 (0.944-0.992) | - |  | - |

^*^The percentage of the average change in rates per year is obtained by subtracting the incidence rate ratio from 1.

†Adjusted for age and sex

‡Adjusted for age, year, body mass index, diabetes, serological status, and smoking status.

IRR, incidence rate ratio; CI, confidence interval.

**Supplementary Table 4: Sensitivity analysis: Standardized event ratios comparing stroke events in rheumatoid arthritis (RA) patients with the comparison cohort, baseline defined as 1 year after RA diagnosis.**

|  | **1972-1998** | **1999-2007** | **2008-2013** |
| --- | --- | --- | --- |
| All | 1.24 (1.04-1.49) | 1.13 (0.88-1.45) | 1.22 (0.80-1.88) |
| Men | 1.22 (0.88-1.68) | 0.97 (0.64-1.48) | 1.66 (0.97-2.85) |
| Women | 1.25 (1.02-1.56) | 1.25 (0.92-1.69) | 0.86 (0.44-1.72) |
| Age < 60 | 0.89 (0.52-1.54) | 1.50 (0.83-2.70) | 0.91 (0.31-2.69) |
| Age > 60 | 1.29 (1.07-1.55) | 1.08 (0.82-1.42) | 1.29 (0.81-2.06) |
| ACR/EULAR criteria | 1.20 (0.98-1.47) | 1.19 (0.91-1.56) | 1.41 (0.92-2.15) |
| RF/ACPA positive | 1.22 (0.96-1.54) | 1.15 (0.69-1.96) | 1.16 (0.69-1.96) |
| Radiographic erosions | 1.31 (1.09-1.56) | 1.43 (0.99-2.08) | 0.92 (0.42-2.01) |

* Stroke events and follow-up time started from 1^st^ of January the year after RA diagnosis. Only inhabitants of Hordaland, Norway were included in the estimates. Point estimates are given with 95% robust confidence intervals. Separate estimates were calculated for the entire RA cohort and 6 RA subgroups defined by age, sex, positive rheumatoid factor or ACPA, fulfilment of the 2010 ACR/EULAR criteria, and radiographic erosions during follow-up. Recurrent events were counted if more than 28 days between discharge to next admission.

ACPA, anti-citrullinated protein antibodies; ACR, American College of Rheumatology; EULAR, European Alliance of Associations for Rheumatology; RA, rheumatoid arthritis; RF, rheumatoid factor; SER, standardized event ratio.
